# Intravenous Tranexamic Acid Reduces Blood Loss and Transfusion Volume in Scoliosis Surgery for Spinal Muscular Atrophy: Results of a 20-Year Retrospective Analysis

**DOI:** 10.3390/ijerph18199959

**Published:** 2021-09-22

**Authors:** Shih-Hsiang Chou, Sung-Yen Lin, Meng-Huang Wu, Yin-Chun Tien, Yuh-Jyh Jong, Wen-Chen Liang, Yen-Mou Lu, Chia-Lung Shih, Cheng-Chang Lu

**Affiliations:** 1 Department of Orthopaedic Surgery, Kaohsiung Medical University Hospital, Kaohsiung 807, Taiwan; stanelychou@gmail.com (S.-H.C.); tony8501031@gmail.com (S.-Y.L.); d740113@cc.kmu.edu.tw (Y.-C.T.); enmol@msn.com (Y.-M.L.); 2 Orthopaedic Research Centre, Kaohsiung Medical University, Kaohsiung 807, Taiwan; 3 Regenerative Medicine and Cell Therapy Research Center, Kaohsiung Medical University, Kaohsiung 807, Taiwan; 4 Graduate Institute of Medicine, College of Medicine, Kaohsiung Medical University, Kaohsiung 807, Taiwan; 5 Department of Orthopaedics, School of Medicine, College of Medicine, Kaohsiung Medical University, Kaohsiung 807, Taiwan; 6 Department of Orthopedics, Taipei Medical University Hospital, Taipei 11031, Taiwan; maxwutmu@gmail.com; 7 Department of Orthopaedics, School of Medicine, College of Medicine, Taipei Medical University, Taipei 11031, Taiwan; 8 Department of Pediatrics, Kaohsiung Medical University Hospital, Kaohsiung 807, Taiwan; yjjong@gap.kmu.edu.tw (Y.-J.J.); peggieliang@yahoo.com.tw (W.-C.L.); 9 Department of Pediatrics, School of Medicine, College of Medicine, Kaohsiung Medical University, Kaohsiung 807, Taiwan; 10 Graduate Institute of Clinical Medicine, College of Medicine, Kaohsiung Medical University, Kaohsiung 807, Taiwan; 11 Department of Laboratory Medicine, Kaohsiung Medical University Hospital, Kaohsiung 807, Taiwan; 12 Clinical Medicine Research Center, Ditmanson Medical Foundation Chia-Yi Christian Hospital, Chia-Yi City 600, Taiwan; stone770116@gmail.com; 13 Department of Orthopaedics, Kaohsiung Municipal Siaogang Hospital, Kaohsiung Medical University, Kaohsiung 812, Taiwan

**Keywords:** blood loss, scoliosis surgery, spinal muscular atrophy, tranexamic acid, transfusion volume

## Abstract

Intravenous tranexamic acid (TXA) has been administered to reduce intraoperative blood loss in scoliosis surgery. However, the therapeutic effect of TXA on spinal muscular atrophy (SMA) scoliosis surgery is not well demonstrated. Therefore, this study aimed to assess the efficacy of intravenous TXA in SMA scoliosis surgery. From December 1993 to August 2020, 30 SMA patients who underwent scoliosis surgery (posterior fusion with fusion level of thoracic second or third to pelvis) were retrospectively enrolled and divided into the TXA group and non-TXA (control) group, with 15 patients in each group. Survey parameters were the amount of blood loss, blood transfusion, crystalloid transfusion volume, intubation time, and associated pulmonary complications (including pneumonia, pulmonary edema, and pulmonary atelectasis). The TXA group had significantly lesser blood loss than the control group (*p* = 0.011). Compared with the control group, the TXA group had significantly lower blood transfusion (*p* < 0.001), crystalloid volume (*p* = 0.041), and total transfusion volume (*p* = 0.005). In addition, the TXA group had fewer postoperative pulmonary complications, and patients with pulmonary complications were associated with a higher relative crystalloid volume and relative total transfusion volume (*p* = 0.003 and 0.022, respectively). In conclusion, TXA can be effective in reducing intraoperative blood loss and crystalloid fluid transfusions during scoliosis surgery in SMA patients, which may aid in reducing postoperative pulmonary complications.

## 1. Introduction

Spinal muscular atrophy (SMA) is an autosomal recessive neuromuscular disorder characterized by spinal motor neuron degeneration resulting in generalized muscle atrophy and weakness [1]. Scoliosis is one of the main clinical problems in neuromuscular diseases, especially in SMA [2,3]. SMA patients could develop a large curve well before peak height growth [4]. In teenage years, SMA patients often have substantial progression of the major curve, with a mean Cobb angle up to 80 degrees [4]. SMA scoliosis would affect the patient’s sitting ability, distorting spinal balance, and further impairing pulmonary function [3]. Surgical interventions aim to correct spinal deformity and halt scoliosis curve progression and pulmonary function decline [5]. Patients undergoing spinal surgery for neuromuscular scoliosis have an increased risk of perioperative bleeding [3,4,6,7,8,9], with the risk of losing >50% of the total blood volume [10], and are strongly associated with an increased requirement for intraoperative fluid and blood transfusion and subsequent pulmonary complication rates [11,12]. Hence, how to decrease the perioperative blood loss and the need for blood and fluid transfusion in SMA patients undergoing scoliosis surgery is a critical concern. 

Tranexamic acid (TXA) is a synthetic analog of the amino acid lysine and a fibrinolysis inhibitor that prevents the binding of plasminogen to the fibrin surface [13]. Perioperative intravenous TXA treatment has demonstrated its effectiveness to reduce blood loss and the amount of blood transfused in orthopedic surgery [14,15]. Prior studies have administered TXA to reduce intraoperative blood loss in the treatment of adolescent idiopathic scoliosis [16,17] and neuromuscular diseases, such as Duchenne muscular dystrophy and cerebral palsy scoliosis [8,9,15,18,19]. However, the effect of TXA administration on SMA scoliosis surgery is still unclear because of the diagnostic heterogeneity and the limited number of SMA patients. Our group has performed scoliosis surgery in SMA patients for more than 20 years, and we administered TXA to reduce intraoperative bleeding in this population since 2009. 

To the best of our knowledge, no study has investigated the effectiveness of intravenous TXA in scoliosis surgery in SMA patients. Therefore, this study aimed to assess the efficacy of intravenous TXA in SMA scoliosis surgery by monitoring blood loss, the volume of blood transfused, and rate of postoperative complications. 

## 2. Materials and Methods

### 2.1. Participants

After receiving Institutional Review Board approval (KMUHIRB-E(I)-20210100), we retrospectively reviewed the medical records of patients surgically treated for scoliosis between December 1993 and August 2020. The diagnosis of spinal muscular atrophy was confirmed by survival motor neuron (SMN) gene deletion and neuropathic change in muscle biopsy. Each patient was first taken care of at pediatric, rehabilitation, and orthopedic departments when the diagnosis of SMA disease was confirmed. We included patients who underwent posterior fusion from the second or third thoracic vertebra down to the pelvis. Exclusion criteria were as follows: patients who received surgery at beyond average age, patients with fusion levels behind the previous stated standard levels, and patients receiving robot-assisted surgery, since there would be a different surgical setting and surgical time. The operative procedures were performed by two senior surgeons (YCT and YML). Patients were divided into two groups: those who received intraoperative TXA treatment (TXA group) and those without intraoperative TXA treatment (control group). In this study cohort, no hemostatic agents other than TXA, such as epsilon-aminocaproic acid (EACA), were used. 

### 2.2. Assessment

All SMA patients were screened for coagulopathy before surgery by measuring serum platelet concentration, prothrombin time (PT), activated partial thrombin time (aPTT), and international normalized ratio (INR). The intraoperative estimated blood loss (EBL) was defined as the total volume of blood loss intraoperatively collected by suction and gauze absorption. In some cases, when a red blood cell salvage system was used, the salvaged blood was counted as intraoperative EBL and the re-infused blood as transfused blood. The percentage of total blood volume lost (TBVL) per patient was calculated from estimated blood loss (numerator) and the estimated total blood volume per patient (denominator). The total blood volume was estimated using 65 mL/kg body weight [20]. Furthermore, we defined the blood transfusion volume (mL) as the sum of the volumes of transfused packed red blood cells and whole blood, the crystalloid infusion volume as the sum of the volumes of intraoperatively infused normal saline and Lactated Ringer’s solution, and the total transfusion volume as the sum of the blood transfusion volume and crystalloid infusion volume. At our institute, one unit of allogeneic packed red blood cells and whole blood was 125 and 250 mL, respectively. For comparison, volume was divided by body weight to obtain the relative volume (mL/kg body weight). The transfusion trigger was similar for both groups and based on a restrictive transfusion threshold (commonly, a hemoglobin level of 7 to 8 g/dL). The surgical time was defined as the time from skin incision to completion of wound closure. In this study, we reported the intubation time and postoperative complications, including suspected myocardial infarction, stroke, venous thromboembolism, pulmonary complications (pneumonia, pulmonary edema, and pulmonary atelectasis), and wound infection. 

### 2.3. Surgical Procedure

**Figure 1:**
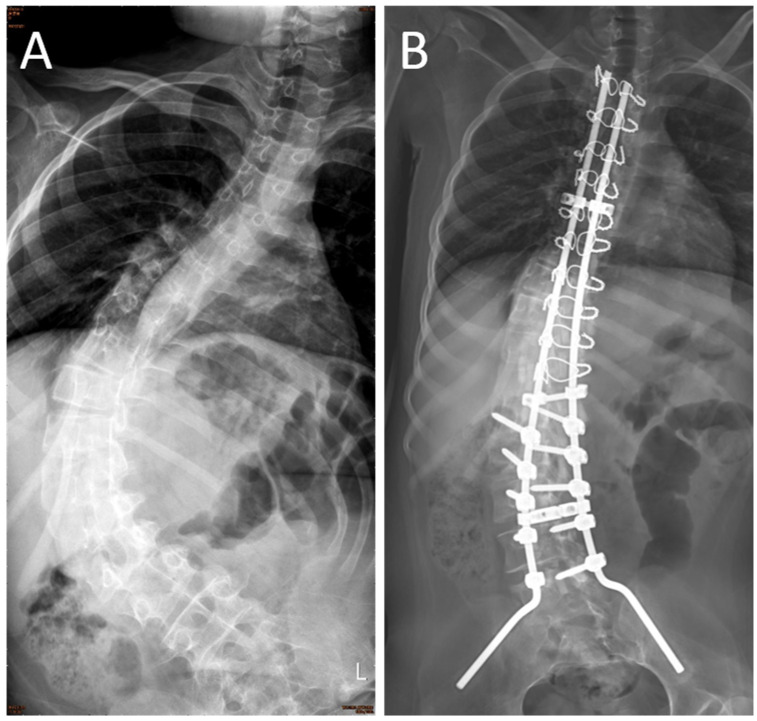
Massive scoliosis curve and pelvic tilt were the characteristics of the preoperative radiogram in spinal muscular atrophy (SMA) patients (**A**). The hybrid technique (including lumbar pedicle screws and thoracic sublaminar wires) combined with pelvic Galveston fixation method was performed for correction and fusion. The postoperative radiogram showed good correction in Cobb’s angle and pelvic tilt (**B**).

The surgical indications of SMA scoliosis included pulmonary function deterioration, progressive scoliosis (major curve angle > 40°), and sitting discomfort. Patients in both groups went through the same technique and procedure. The hybrid technique (including lumbar pedicle screws and thoracic sublaminar wires) combined with the pelvic Galveston fixation method was performed for correction and fusion (Figure 1). The fusion levels included the second or third thoracic vertebra down to the pelvis. The bone graft was obtained from autogenous bone (harvested by laminotomy) and artificial bone, which was composed of hydroxyapatite and β-tricalcium phosphate. Before skin wound closure, the area was electrocauterized for hemostasis, and a Hemovac drain (PAHSCO; Pacific Hospital Supply CO., LTD, Miaoli, Taiwan) was inserted. 

### 2.4. Postoperative Course

All patients were sent postoperatively to the pediatric intensive care unit (PICU) for further care, which was managed by the same care team. The intubation time was defined as the period from the end of surgery to endotracheal tube removal in the PICU. The Hemovac tube was kept and was allowed to be removed when daily amount of fluid was less than 100 mL. The intraoperative assessment was analyzed to determine the risk factors of postoperative complications (ex: pulmonary complications). The significant operative variables were assumed as a reference to analyze the relationship between TXA administration and postoperative complications.

### 2.5. Drug Dose

Since 2009, SMA patients who underwent scoliosis correction surgery have received TXA. The TXA group received TXA with an intravenous loading dose of 100 mg/kg, followed by a maintenance dose of 10 mg/kg/h until skin closure. This high-dose TXA protocol was selected based on a previous study about neuromuscular scoliosis [18].

### 2.6. Statistical Analysis

Data were analyzed using standardized statistical software (Statistical Package for Social Science; version 19.0; SPSS, Inc., Chicago, IL, USA). Statistical analysis was descriptive (mean and standard deviation) for each parameter. Normality was tested using the Shapiro–Wilk test. If both groups’ tests indicate normal distribution, the independent sample *t*-test was adopted. Otherwise, the Mann–Whitney U test was adopted. Data of categorical variables were analyzed using the Chi-Square test to compare the control group with that of the TXA group. To determine the impact factors of developing postoperative pulmonary complications, discrete data between subgroups were analyzed by the Chi-Square test. *p*-values less than 0.05 were considered significant. 

## 3. Results

A total of 57 SMA patients undergoing corrective surgery during these periods were recruited in this study. One patient due to beyond average age of receiving surgery (when she was at forty-two years old), five patients due to not reaching standard long fusion levels (upper instrumented vertebrae should be upper than fourth thoracic vertebrae), and twenty-one patients due to receiving robot-assisted surgery were excluded from this study. The 30 SMA patients were distributed to the TXA group (*n* = 15) and control group (*n* = 15). The frequency of the SMA subtype in these two groups was identical (SMA type II:SMA type III = 12:3). No significant differences in preoperative demographic data, preoperative test results (including preoperative hematocrit (%), serum platelet concentration, PT, aPTT, and INR), preoperative major curve angle, and postoperative data, including major curve angle, correction angle, correction rate, and surgical time, were found between the two groups (Table 1).

### 3.1. Intraoperative Blood Loss

Compared with the control group, the TXA group showed lower estimated blood loss (EBL) without significant difference. The TXA group had a statistically lower average TBVL compared to that in the control group (*p* < 0.05; Table 2). The mean TBVL for the TXA group (52.1 ± 17.8 (%)) was 49% that of the control group (106.7 ± 95.0 (%)); in other words, TXA administration decreased the mean rate of TBVL by 51%.

### 3.2. Intraoperative Blood and Fluid Transfusion Volume

The volume of blood transfusion in the TXA group (498.7 mL) was significantly lower than that in the control group (1250 mL) (*p <* 0.001). Crystalloid volume (*p* = 0.041) and total transfusion volume (*p* = 0.005) were significantly lower in the TXA group than in the control group (Table 2). The mean blood transfusion, crystalloid, and total transfusion volumes in the TXA group were 40%, 74%, and 65% of their respective volumes in the control group, representing a decrease of 60%, 26%, and 35% in the blood transfusion, crystalloid, and total transfusion volumes, respectively. 

### 3.3. Intubation Time and Pulmonary Complication Rate

The intubation time was non-significantly shorter in the TXA group than in the control group (*p* = 0.106; Table 2). The postoperative complications comprised of pulmonary associated (*n* = 13) as the majority, and the rare complication of wound infection (*n* = 1). No intravenous thrombosis events occurred in either group. In the comparison of the effect of TXA on pulmonary complications, the TXA group had a trend of a lower pulmonary complication rate (5/15 = 33.3%) than the control group (8/15 = 53.3%), but no significant between-group differences were found (*p* = 0.269; Table 2). Patients with pulmonary complications (versus those without these complications) were more likely to receive higher relative crystalloid volumes and relative total transfusion volumes (*p* = 0.003, 0.022, respectively; Table 3). 

### 3.4. Crystalloid Volume Overload and Pulmonary Complications

**Figure 2:**
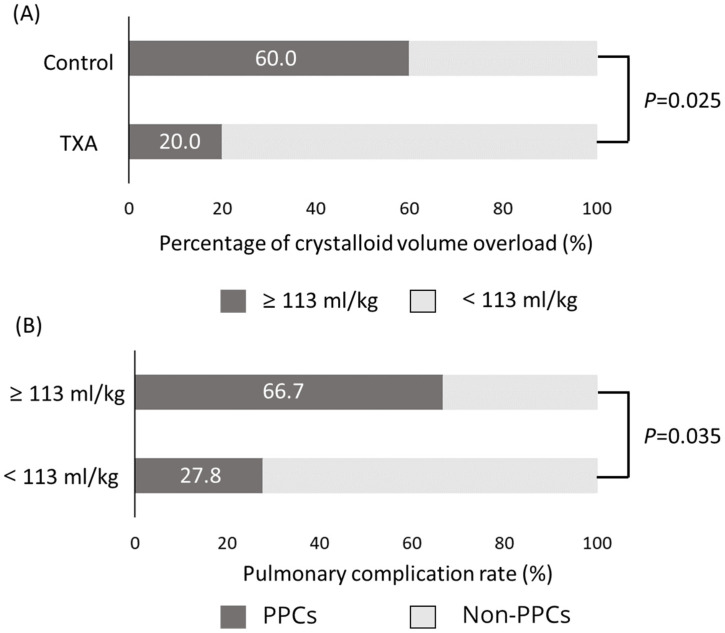
Proportion of patients with crystalloid volume overload (≥113 mL/kg) in the control and tranexamic acid (TXA) groups (**A**). Proportion of patients with pulmonary complications (PPCs) in the crystalloid volume overload (≥113 mL/kg) and non-overload (<113 mL/kg) groups (**B**).

Considering the relationship between crystalloid volume overload and pulmonary complications, we set 113 mL/kg as the reference level of crystalloid volume transfused to analyze the relationship among TXA administration, crystalloid volume, and pulmonary complications. The reference value of 113 mL/kg was chosen because the average intraoperative crystalloid volume consumption in our study was 113.1 ± 53.6 mL/kg. The following discrete data between groups were analyzed by the Chi-Square test. The TXA group had a significantly lower incidence of crystalloid volume overload than the control group (*p* = 0.025) (Figure 2A). Moreover, the pulmonary complication rate was significantly higher in the crystalloid volume overload group than in the non-overload group (*p* = 0.035; Figure 2B). To understand the difference of crystalloid overload impact on these two groups, we further performed the stratifying analysis of patients from control and TXA groups into fluid overload and non-overload subgroups. There was a trend of higher incidence to develop pulmonary complications in the fluid overload subgroup of both the control and TXA groups without a statistically significant difference (*p* = 0.205 and 0.171, respectively; Supplementary Figure S1).

## 4. Discussion

This study assessed the effect of intravenous TXA administration on SMA scoliosis surgery with regard to estimated blood loss, intraoperative transfusion, and postoperative complications. To the best of our knowledge, this is the first study to investigate the effectiveness of intravenous TXA in scoliosis surgery in SMA patients. We demonstrated that intravenous TXA was safe and effective in reducing intraoperative blood loss by 51%, the volume of blood transfusion by 60%, crystalloid volume by 26%, and total transfusion volume by 35% compared with the control group. Compared with the TXA group, the control group had a trend of higher intraoperative crystalloid volume and postoperative pulmonary complications, although no significant difference was found. The study results indicated that the administration of TXA during scoliosis surgery in SMA patients can decrease intraoperative bleeding, reduce the transfusion volume and the associated pulmonary complications, and thereby improve the patient’s safety. 

Massive hemorrhage, which is defined as the loss of >50% of the TBVL, is a major complication in scoliosis surgery of neuromuscular patients (e.g., Duchenne muscular dystrophy, cerebral palsy, SMA) [3,6,7,8,9]. The mechanism of extensive bleeding during neuromuscular scoliosis surgery is not conclusive if multiple factors are considered, such as involvement of more spinal segments in fusion, decreased coagulation factor reserve with poor platelet quality, changes in the mitochondrial structure of the vascular smooth muscles, and increased fibrinolytic activity [3]. To investigate the amount of blood loss in the neuromuscular scoliosis surgery, Edler et al. retrospectively analyzed 163 patients who underwent scoliosis surgery and subclassified them into neuromuscular and non-neuromuscular groups [10]. They found that the perioperative blood loss was up to seven times higher in patients with neuromuscular scoliosis than in those with non-neuromuscular scoliosis. To date, only a few studies have evaluated the effectiveness of TXA administration on reducing intraoperative blood loss during neuromuscular scoliosis surgery (Table 4) [8,15,18,19]. In the present study, the percentage of total blood volume lost in the TXA group was 49% lower than that in the control group (*p* < 0.05). The results indicated that intraoperative TXA administration can effectively decrease the intraoperative and total blood loss in SMA patients undergoing scoliosis surgery. 

Neuromuscular scoliosis surgery is a risk for massive perioperative blood loss and may sequentially require massive amounts of both fluid infusion and blood transfusion [3,6,8,9,11]. Meert et al. found that neuromuscular scoliosis patients are 7.8 times more likely to receive an allogeneic transfusion than idiopathic scoliosis patients [21]. The transfusion of blood products (such as donor-directed or allogeneic) adds to healthcare costs and increases the risk of complications, such as dilutional coagulopathy, pulmonary edema, and surgical site infection [7,22]. Furthermore, the updated concept of damage control orthopedics (DCO) has emerged and guided new strategies in preventing massive bleeding-induced coagulopathy [23]. The role of preoperative and intraoperative TXA utilizing has demonstrated its significant impact on morbidity and mortality. In addition to early adoption of TXA, high platelet or plasma to RBC transfusion ratios were associated with a decreased need for massive transfusion and increased survival in injured patients with bleeding [24]. Therefore, it is important to understand the effect of TXA on SMA patients undergoing scoliosis surgery. Shapiro et al. found that TXA administration in patients undergoing Duchenne scoliosis surgery can decrease blood transfusion volume by 46% compared to control patients [18]. Dhawale et al. compared the effects of using different antifibrinolytics (including TXA and EACA) in patients with cerebral palsy who underwent scoliosis surgery [8]. They demonstrated that TXA administration (versus EACA and control) further decreased cell salvage transfusion volume. In our study, TXA tended to reduce blood transfusion volume during SMA scoliosis surgery. Furthermore, our study results showed that TXA admission can significantly (*p* < 0.05) reduce intraoperative crystalloid volume and total transfusion volume in SMA patients undergoing scoliosis surgery. 

During general surgery, a large amount of intraoperative fluid administration would predispose patients to pulmonary complications, such as pulmonary edema, mechanical ventilation demand, and pneumonia [25,26,27]. In addition, Berman et al. found that the massive total crystalloid or blood volume administered correlated with delay extubation in multilevel spine surgery [28]. Since massive blood loss during surgery for SMA scoliosis is possible, substantive intraoperative intravenous fluid administration is inevitable [29]. Up to 50% of patients who undergo surgery for SMA scoliosis develop pulmonary complications and need long-term respiratory support in the postoperative period [3]. In this study, our calculation of the crystalloid volume was based on the consumption of normal saline and Lactated Ringer’s because they are the recommended first-line resuscitation fluids in patients undergoing surgery [27]. The significant reduction in physiological blood loss by TXA lowered the risk of developing intravenous crystalloid overload and enabled postoperative extubation much earlier. In our study, we demonstrated that intravenous crystalloid overload increased the risk of postoperative pulmonary complications. In the control group, SMA patients presented a higher incidence of crystalloid volume overload and might develop more pulmonary complications after scoliosis surgery. Our results indicated that intraoperative treatment with TXA reliably prevented postoperative pulmonary complications by reducing blood loss and crystalloid consumption and thereby the possibility of crystalloid overload. 

This study had limitations. First, this was a 20-year retrospective cohort study, and patients in the control group were all recruited early in the study. The new care concept and facility in managing perioperative blood loss might also affect the result of comparison between TXA and control groups. Second, we were unable to discuss coagulopathy status at the end of the operation and postoperative period because of the lack of laboratory data, e.g., INR, PT, aPTT, and platelet count. Third, the sample size was small even with the 20 years of collections and chart review. Finally, the limited case number reduced the statistical power. Further prospective studies with a larger sample population or multiple center studies should be conducted to confirm the findings of this study. 

## 5. Conclusions

In conclusion, this study suggests that TXA can effectively decrease the intraoperative blood loss and crystalloid transfusion volume during posterior spinal fusion for SMA scoliosis. Furthermore, TXA administration in SMA patients undergoing scoliosis surgery can reduce the incidence of crystalloid fluid overload, allow earlier postoperative extubation, and thereby reduce postoperative pulmonary complications.

## Figures and Tables

**Table 1:** Demographic data and surgical profile.

	Control Group (*n* = 15)	TXA Group (*n* = 15)	*p*-Value
Age (years)	14.5 ± 5.5	12.7 ± 5.6	0.233 ^a^
Sex (Female: Man) SMA II:SMAIII	9:6 12:3	10:5 12:3	0.583 ^a^ 1.000 ^a^
Height (cm)	148.6 ± 14.2	150.1 ± 12.6	0.769 ^a^
Weight (kg)	33.1 ± 8.6	39.6 ± 12.8	0.171 ^a^
Hematocrit (%)	39.4 ± 3.2	41.7 ± 3.5	0.067 ^b^
Coagulation profile			
Platelet (×10^3^)	313.7 ± 72.3	321.9 ± 58.3	0.735 ^b^
PT (Sec)	11.6 ± 0.7	11.0 ± 1.0	0.071 ^b^
aPTT (Sec)	31.9 ± 4.5	29.5 ± 2.4	0.077 ^b^
INR	0.96 ± 0.08	1.00 ± 0.06	0.128 ^b^
Scoliosis major curve			
Preoperative Cobb’s	77.1 ± 29.1	70.3 ± 22.8	0.595 ^a^
Postoperative Cobb’s	29.6 ± 19.6	22.0 ± 14.2	0.250 ^a^
Correction (°)	46.3 ± 14.5	48.3 ± 14.0	0.567 ^a^
Correction rate (%)	60.8 ± 14.0	70.0 ± 12.9	0.072 ^b^
Surgical time (h)	9.0 ± 1.6	8.7 ± 1.3	0.461 ^a^

a: Mann–Whitney U test; b: independent sample *t*-test; TXA, tranexamic acid; PT, prothrombin time; aPTT, activated partial thrombin time; INR, international normalized ratio.

**Table 2:** Comparison of estimated blood loss, intraoperative infusion, and postoperative care between the Tranexamic acid group and control group.

	Control Group (*n* = 15)	TXA Group (*n* = 15)	*p*-Value
EBL (mL)	2023.8 ± 1673.4	1327.0 ± 685.2	0.174
TBVL (%)	106.7 ± 95.0	52.1 ± 17.8	0.011 *
Intraoperative supplement			
Blood transfusion			
volume (mL)	1250.0 ± 1052.2	498.7 ± 158.4	<0.001 *
relative volume (mL/kg)	43.3 ± 40.5	13.4 ± 4.2	<0.001 *
Crystalloid			
volume (mL)	4154.0 ± 1530.9	3058.0 ± 836.2	0.041 *
relative volume (mL/kg)	144.1 ± 57.5	82.2 ± 24.4	<0.001 *
Total transfusion			
volume (mL)	5404.0 ± 2308.5	3523.4 ± 890.2	0.005 *
relative volume (mL/kg)	187.4 ± 91.2	95.2 ± 26.4	<0.001 *
Postoperative care			
Intubation time (h)	52.8 ± 58.5	19.1 ± 12.9	0.106
Postoperative complications			
Wound infection	0	1	
VTE	0	0	
Pulmonary complications	8 (8/15 = 53.3%)	5 (5/15 = 33.3%)	0.269
*Pneumonia + pulmonary edema*	6	2 ^#^	
* Pulmonary edema*	1	2	
* Atelectasis*	1	1	

All continuous variables were tested by Mann–Whitney U test; TXA, tranexamic acid; EBL, estimated blood loss; TBVL, percentage of total blood volume lost; VTE, venous thromboembolism; # one patient underwent reintubation due to respiratory failure; *, *p* < 0.05.

**Table 3:** Comparison of operative variables between postoperative pulmonary complication group and non-pulmonary complication group.

	Pulmonary Complication (*n* = 13)	Non-Pulmonary Complication (*n* = 17)	*p*-Value
EBL (mL)	1688.62 ± 1596.0	1665.3 ± 1085.4	0.869
TBVL (%)	95.2 ± 103.5	67.3 ± 34.4	0.680
Intraoperative supplement			
Crystalloid			
volume (mL)	4109.2 ± 1578.4	3221.2 ± 997.0	0.113
relative volume (mL/kg)	142.2 ± 62.7	90.9 ± 32.4	0.003 *
Total transfusion			
volume (mL)	4987.2 ± 2507.3	4065.6 ± 1382.4	0.263
relative volume (mL/kg)	176.6 ± 103.7	114.3 ± 44.9	0.022 *
Postoperative care			
Intubation time (h)	57.5 ± 61.6	19.4 ± 12.5	0.183

All continuous variables were tested by Mann–Whitney U test; EBL: intraoperative estimated blood loss; TBVL: percentage of total blood volume lost; *, *p* < 0.05.

**Table 4:** Tranexamic acid-treated patients with neuromuscular scoliosis reported in previous studies and this study.

	This Study	Neilipovitz et al. [15]	Sethna et al. [19]	Shapiro et al. [18]	Dhawale et al. [8]
Disease	SMA	miscellaneous	miscellaneous	DMD	CP
Number(s)	30	unknown	22	56	70
Reduced blood loss	51% *	10%	48% *	58% *	51.5% *
Reduced blood transfusion	60% *	30% *	42% *	46% *	12.5%
Reduced crystalloid volume	26% *	12.3%	undescribed	undescribed	undescribed

SMA, spinal muscular atrophy; DMD, Duchenne muscular dystrophy; CP, cerebral palsy; * significant differences between TXA and control groups (*p* < 0.05).

## Data Availability

Data supporting reported results can be requested from the first author.

## Supplementary Materials

The following are available online at www.mdpi.com/article/10.3390/ijerph18199959/s1, Figure S1: The impact of crystalloid fluid overload on control and TXA groups.

## References

[1] Lunn M.R., Wang C.H. (2008). Spinal muscular atrophy. Lancet.

[2] Piazzolla A., Solarino G., De Giorgi S., Mori C.M., Moretti L., De Giorgi G. (2011). Cotrel-Dubousset instrumentation in neuromuscular scoliosis. Eur. Spine J..

[3] Garg S. (2016). Management of scoliosis in patients with Duchenne muscular dystrophy and spinal muscular atrophy: A literature review. J. Pediatr. Rehabil. Med..

[4] Brooks J.T., Sponseller P.D. (2016). What’s new in the management of neuromuscular scoliosis. J. Pediatr. Orthop..

[5] Chou S.H., Lin G.T., Shen P.C., Lue Y.J., Lu C.C., Tien Y.C., Lu Y.M. (2017). The effect of scoliosis surgery on pulmonary function in spinal muscular atrophy type II patients. Eur. Spine J..

[6] Shapiro F., Sethna N. (2004). Blood loss in pediatric spine surgery. Eur. Spine J..

[7] Kannan S., Meert K.L., Mooney J.F., Hillman-Wiseman C., Warrier I. (2002). Bleeding and coagulation changes during spinal fusion surgery: A comparison of neuromuscular and idiopathic scoliosis patients. Pediatr. Crit. Care Med..

[8] Dhawale A.A., Shah S.A., Sponseller P.D., Bastrom T., Neiss G., Yorgova P., Newton P.O., Yaszay B., Abel M.F., Shufflebarger H. (2012). Are antifibrinolytics helpful in decreasing blood loss and transfusions during spinal fusion surgery in children with cerebral palsy scoliosis?. Spine (Phila Pa 1976).

[9] McLeod L.M., French B., Flynn J.M., Dormans J.P., Keren R. (2015). Antifibrinolytic use and blood transfusions in pediatric scoliosis surgeries performed at US children's hospitals. J. Spinal Disord. Tech..

[10] Edler A., Murray D.J., Forbes R.B. (2003). Blood loss during posterior spinal fusion surgery in patients with neuromuscular disease: Is there an increased risk?. Paediatr. Anaesth..

[11] Jia R., Li N., Xu B.Y., Zhang W., Gu X.P., Ma Z.L. (2017). Incidence, influencing factors, and prognostic impact of intraoperative massive blood loss in adolescents with neuromuscular scoliosis: A STROBE-compliant retrospective observational analysis. Medicine (Baltim.).

[12] Toll B.J., Samdani A.F., Janjua M.B., Gandhi S., Pahys J.M., Hwang S.W. (2018). Perioperative complications and risk factors in neuromuscular scoliosis surgery. J. Neurosurg. Pediatr..

[13] Mannucci P.M. (1998). Hemostatic drugs. N. Engl. J. Med..

[14] Yang B., Li H., Wang D., He X., Zhang C., Yang P. (2013). Systematic review and meta-analysis of perioperative intravenous tranexamic acid use in spinal surgery. PLoS ONE.

[15] Neilipovitz D.T., Murto K., Hall L., Barrowman N.J., Splinter W.M. (2001). A randomized trial of tranexamic acid to reduce blood transfusion for scoliosis surgery. Anesth. Analg..

[16] Lykissas M.G., Crawford A.H., Chan G., Aronson L.A., Al-Sayyad M.J. (2013). The effect of tranexamic acid in blood loss and transfusion volume in adolescent idiopathic scoliosis surgery: A single-surgeon experience. J. Child. Orthop..

[17] Verma K., Errico T., Diefenbach C., Hoelscher C., Peters A., Dryer J., Huncke T., Boenigk K., Lonner B.S. (2014). The relative efficacy of antifibrinolytics in adolescent idiopathic scoliosis: A prospective randomized trial. J. Bone Joint Surg. Am..

[18] Shapiro F., Zurakowski D., Sethna N.F. (2007). Tranexamic acid diminishes intraoperative blood loss and transfusion in spinal fusions for duchenne muscular dystrophy scoliosis. Spine (Phila Pa 1976).

[19] Sethna N.F., Zurakowski D., Brustowicz R.M., Bacsik J., Sullivan L.J., Shapiro F. (2005). Tranexamic acid reduces intraoperative blood loss in pediatric patients undergoing scoliosis surgery. Anesthesiology.

[20] Brecher M.E., Monk T., Goodnough L.T. (1997). A standardized method for calculating blood loss. Transfusion.

[21] Meert K.L., Kannan S., Mooney J.F. (2002). Predictors of red cell transfusion in children and adolescents undergoing spinal fusion surgery. Spine (Phila Pa 1976).

[22] Schuster J.M., Rechtine G., Norvell D.C., Dettori J.R. (2010). The influence of perioperative risk factors and therapeutic interventions on infection rates after spine surgery: A systematic review. Spine (Phila Pa 1976).

[23] Leighton J.L., You D., Schneider P. (2020). Limiting blood loss in orthopaedic trauma. Strategies and effect. Injury.

[24] Balvers K., van Dieren S., Basksaas-Asen K., Gaarder C., Brohi K., Eaglestone S., Stanworth S., Johansson P.J., Ostrowski S.R., Stensballe J. (2017). Combined effect of therapeutic strategies for bleeding injury on early survival, transfusion needs and correction of coagulopathy. Br. J. Surg..

[25] Schrier R.W., Wang W. (2004). Acute renal failure and sepsis. N. Engl. J. Med..

[26] Cordemans C., De Laet I., Van Regenmortel N., Schoonheydt K., Dits H., Huber W., Malbrain M.L. (2012). Fluid management in critically ill patients: The role of extravascular lung water, abdominal hypertension, capillary leak, and fluid balance. Ann. Intensive Care.

[27] Myburgh J.A., Mythen M.G. (2013). Resuscitation fluids. N. Engl. J. Med..

[28] Anastasian Z.H., Gaudet J.G., Levitt L.C., Mergeche J.L., Heyer E.J., Berman M.F. (2014). Factors that correlate with the decision to delay extubation after multilevel prone spine surgery. J. Neurosurg. Anesthesiol..

[29] Yu X., Xiao H., Wang R., Huang Y. (2013). Prediction of massive blood loss in scoliosis surgery from preoperative variables. Spine (Phila Pa 1976).

