# Supplementary material for: Intravenous Tranexamic Acid Reduces Blood Loss and Transfusion Volume in Scoliosis Surgery for Spinal Muscular Atrophy: Results of a 20-Year Retrospective Analysis
Source: Int J Environ Res Public Health. 2021 Sep 22;18(19):9959. doi: 10.3390/ijerph18199959 (PMC8507662; doi:10.3390/ijerph18199959)
Supplement: Supplementary file 1 [file ijerph-18-09959-s001.zip › ijerph-1339632-supplementary.pdf]

Supplementary Material

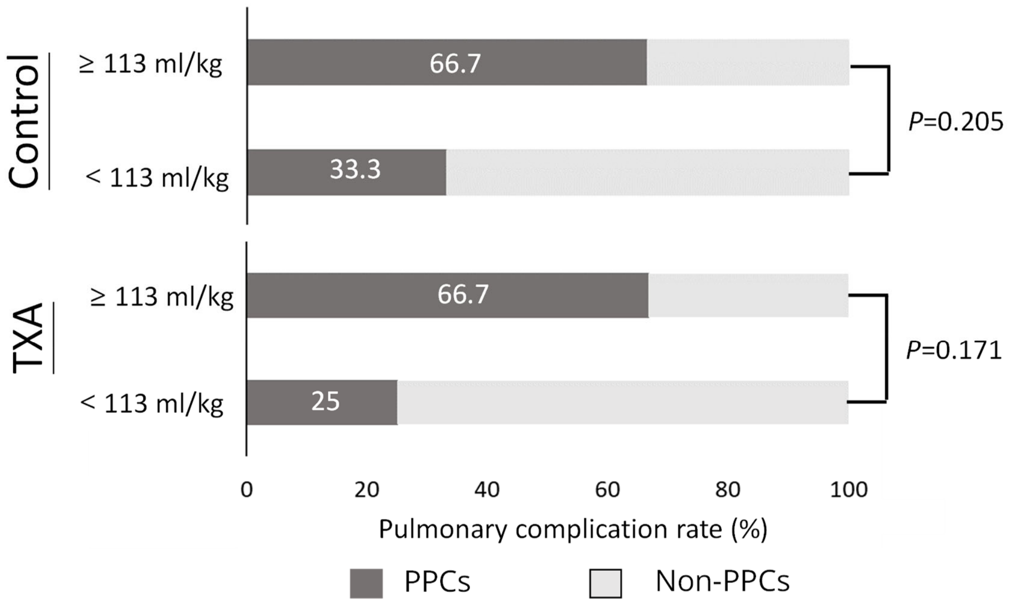

Figure S1. Percentage of patients with pulmonary complications (PPCs) between crystalloid volume overload (≥113 ml/kg) and non-overload (<113 ml/kg) subgroups in the control (upper side) and tranexamic acid (TXA) (lower side) groups, respectively.
